# Whole-genome sequencing facilitates patient-specific quantitative PCR-based minimal residual disease monitoring in acute lymphoblastic leukaemia, neuroblastoma and Ewing sarcoma

**DOI:** 10.1038/s41416-021-01538-z

**Published:** 2021-09-01

**Authors:** Vinod Vijay Subhash, Libby Huang, Alvin Kamili, Marie Wong, Dan Chen, Nicola C. Venn, Caroline Atkinson, Chelsea Mayoh, Pooja Venkat, Vanessa Tyrrell, Glenn M. Marshall, Mark J. Cowley, Paul G. Ekert, Murray D. Norris, Michelle Haber, Michelle J. Henderson, Rosemary Sutton, Jamie I. Fletcher, Toby N. Trahair

**Affiliations:** 1 Children’s Cancer Institute, Lowy Cancer Research Centre, UNSW Sydney, Sydney, NSW Australia; 2 School of Women’s and Children’s Health, UNSW Sydney, Sydney, NSW Australia; 3 Kids Cancer Centre, Sydney Children’s Hospital, Randwick, NSW Australia; 4 Murdoch Children’s Research Institute, Royal Children’s Hospital, Parkville, VIC Australia; 5 Peter MacCallum Cancer Centre, Melbourne, VIC Australia; 6 UNSW Centre for Childhood Cancer Research, UNSW Sydney, Sydney, NSW Australia

**Keywords:** Paediatric cancer, Tumour biomarkers, Cancer genomics, Prognostic markers

## Abstract

**Background:**

Minimal residual disease (MRD) measurement is a cornerstone of contemporary acute lymphoblastic leukaemia (ALL) treatment. The presence of immunoglobulin (Ig) and T cell receptor (TCR) gene recombinations in leukaemic clones allows widespread use of patient-specific, DNA-based MRD assays. In contrast, paediatric solid tumour MRD remains experimental and has focussed on generic assays targeting tumour-specific messenger RNA, methylated DNA or microRNA.

**Methods:**

We examined the feasibility of using whole-genome sequencing (WGS) data to design tumour-specific polymerase chain reaction (PCR)-based MRD tests (WGS-MRD) in 18 children with high-risk relapsed cancer, including ALL, high-risk neuroblastoma (HR-NB) and Ewing sarcoma (EWS) (*n* = 6 each).

**Results:**

Sensitive WGS-MRD assays were generated for each patient and allowed quantitation of 1 tumour cell per 10^−4^ (0.01%)–10^–5^ (0.001%) mononuclear cells. In ALL, WGS-MRD and Ig/TCR-MRD were highly concordant. WGS-MRD assays also showed good concordance between quantitative PCR and droplet digital PCR formats. In serial clinical samples, WGS-MRD correlated with disease course. In solid tumours, WGS-MRD assays were more sensitive than RNA-MRD assays.

**Conclusions:**

WGS facilitated the development of patient-specific MRD tests in ALL, HR-NB and EWS with potential clinical utility in monitoring treatment response. WGS data could be used to design patient-specific MRD assays in a broad range of tumours.

## Background

Sensitive treatment response monitoring, using minimal residual disease (MRD) assays, is a core component of contemporary risk and response-adapted treatment programmes for acute lymphoblastic leukaemia (ALL) [1–6]. MRD is used to refine risk stratification and treatment for both newly diagnosed [3, 5, 7] and relapsed patients [8, 9], and aids in the identification of patients who may benefit from more intensive therapy [10–12] or who are at higher risk of disease recurrence [2, 9, 12–15]. MRD monitoring in ALL [16] has been successfully implemented partly because of the relative ease in identifying ALL-specific recombination events in immunoglobulin (Ig) and T cell receptor (TCR) genes and the development and standardisation of quantitative reporting for both flow cytometry and quantitative polymerase chain reaction (qPCR) assays [6, 17–21]. Moreover, MRD tests based on detecting recurrent *IKZF1* gene deletions, translocations involving the *KMT2A* or *MLL* gene and the *BCR-ABL1* gene fusion, which occur in small proportions of B-ALL patients, have been validated [22].

In contrast, developing MRD assays for other paediatric tumours, including neuroblastoma (NB) and Ewing sarcoma (EWS) has been challenging [23, 24]. Approximately 50% of NB patients have high-risk disease (HR-NB) with extensive bone marrow (BM) metastasis at diagnosis [25, 26]. Improvements in the cytological analysis of BM biopsies provide valuable quantitative data at diagnosis [27]. Currently, the international consensus criteria for the quantitative analysis of BM disease in NB involves immunocytology and quantitative reverse transcriptase-PCR (qRT-PCR) [28]. In EWS, ~27% of patients have metastatic disease at diagnosis, and BM involvement is an independent risk factor for poor prognosis [29, 30]. A dynamic assessment of disease burden through stages of diagnosis, treatment and follow-up has the potential to impact treatment strategies and survival outcomes in these cancers.

HR-NB and EWS patients have a high risk of relapse and disease progression after initial treatment [31, 32]. Unlike ALL, where Ig/TCR rearrangements are readily identified in most patients [17, 18], developing patient-specific, DNA-based quantitative PCR MRD (qPCR-MRD) assays in HR-NB and EWS has been limited by the absence of readily identifiable, tumour-specific targets. Instead, MRD detection in these cancers has focussed on the detection of tumour-specific messenger RNA (mRNA) using qRT-PCR of NB-specific RNA markers, such as *TH* and *PHOX2B*, or *EWSR1* gene fusion transcripts with the *ETS* family or *FLI1* gene for EWS-specific markers [33–36].

Of note, the expression of mRNA markers shows high transcript level variability between primary and disseminated sites, and it is unclear whether these transcripts undergo treatment-related changes in expression [37]. Other liquid biopsy approaches have been developed including serial detection of hypermethylated DNA targets [38], exosomal microRNA (miRNA) [39] and, recently, characterisation of the cell-free DNA (cfDNA) in NB and EWS using targeted and untargeted approaches such as droplet digital PCR (ddPCR) and WGS has shown the ability to monitor tumour evolution based on genetic and epigenetic profiles [40, 41]. Also, multiplex-PCR approaches using a panel of mRNAs has shown improved sensitivity of MRD monitoring in NB [42]. The potential utility of these approaches for clinical monitoring of MRD is still to be established. The limited sensitivity of current approaches together with the imprecision of MRD assessment has meant that whilst high levels of detectable MRD at diagnosis are associated with a poor prognosis in NB, a reduction in MRD following chemotherapy does not provide additional predictive information [43]. In EWS, the prognostic relevance of molecularly detectable fusion transcripts remains controversial [44]. Consequently, MRD is not currently used for risk stratification or modification of therapy in HR-NB and EWS patients, although it is used experimentally within clinical trials.

Recent advances in genomics and bioinformatics, allied with reductions in the cost of next-generation sequencing, has facilitated the development of precision medicine programmes and allowed increasing numbers of solid tumours to undergo rapid whole-genome sequencing (WGS). Whilst the primary focus of precision medicine has been the rapid identification of targetable genetic alterations [45], the WGS data can also be used to determine unique tumour-specific gene sequences and to develop quantitative patient-specific MRD assays, independent of tumour type, enabling DNA-based, qPCR MRD assessment for solid tumours.

The purpose of this study was to determine whether individualised, tumour-specific breakpoints identified by WGS of the tumour DNA can serve as reliable targets for MRD detection in paediatric cancers, to assess their sensitivity, and to determine whether MRD levels in the BM and PB can be accurately quantified using this marker. WGS-MRD assays were optimised based on the Euro-MRD guidelines [18] and tested using both qPCR and ddPCR platforms. Assay performance was validated using serially collected clinical specimens and compared directly to existing Ig/TCR or RNA-based assays.

## Methods

### Patients and samples

Patients with relapsed HR-NB (*n* = 6), EWS (*n* = 6) and ALL (*n* = 6) who were enrolled on either the PRecISion Medicine for Children with Cancer (PRISM) study (NCT03336931) or its preceding feasibility pilot study, TARGET [45], were included in this study. The TARGET study was approved by the Sydney Children’s Hospital (SCH) Network Human Research Ethics Committee (LNR/14/SCHN/497) and the PRISM study was approved by the Hunter New England Human Research Ethics Committee (HREC/17/HNE/29). These studies were performed in accordance with the Declaration of Helsinki and informed parental consent was obtained for WGS, the generation of patient-derived xenograft (PDX) models and liquid biopsy research including MRD studies. Briefly, patients with high-risk cancer, defined as having a survival probability of ≤30%, were eligible to participate. Fresh tumour and PB samples from participants were obtained and subjected to genomic analysis, which included WGS (90×) and RNA-sequencing for tumour samples and WGS (30×) for germline DNA [45]. The tumour and germline WGS samples were analysed to identify tumour-specific genetic changes, such as translocations, inversions, duplications, amplifications, deletions and/or insertions, which could be used as potential targets to develop a patient-specific MRD assay. Where sufficient fresh tumour was available, it was inoculated into immunodeficient mice to generate PDXs. These experiments were performed in accordance with the guidelines approved by the University of New South Wales Animal Care and Ethics Committee (ACEC 17/101B) and the requirements of the Australian Code for the Care and Use of Animals for Scientific Purposes. The resulting PDX models were validated against patient tumour DNA as previously described [46]. DNA obtained from patient diagnostic material (ALL) and PDX of diagnostic tumours (HR-NB, EWS) were used for MRD assay development and optimisation. BM and peripheral blood (PB) samples collected from patients during treatment were utilised for MRD assay validation and disease monitoring. For BM and PB samples, mononuclear cells (MNCs) were isolated by density gradient centrifugation using Lymphoprep (Stemcell Technologies) as per the manufacturer’s instructions. All samples were processed within 24 h of collection and MNCs were cryopreserved in 10% dimethyl sulfoxide and stored at –180 °C. Genomic DNA from ALL samples was extracted using NucleoBond CB Kits (Takara Bio). Genomic DNA and RNA were extracted from HR-NB and EWS samples using AllPrep DNA/RNA Mini Kit (Qiagen) as per the manufacturer’s instructions. DNA and RNA samples of pooled PB cells from healthy donors were used as negative controls. The extracted DNA and RNA samples were stored in aliquots at –80 °C until further use. Dissociated PDX cells were utilised for cell spike-in experiments with BM cells obtained from the SCH Bone Marrow Transplant Lab. The viable cell number was determined by the trypan blue exclusion assay.

### Detection of WGS and Ig/TCR DNA-MRD markers

We utilised the PRISM WGS data [45] to identify patient-specific DNA breakpoints from genomic structural variations such as deletions, duplications, translocations and inversions. Structural rearrangements were identified using GRIDSS (v2.7.2) and annotated using Ensembl genes as described previously [45]. For assay development, WGS-MRD targets were selected based on the variant allele frequency (VAF) score, using a VAF threshold of ≥0.3 as per published reports [47, 48]. In HR-NB and EWS, the number of targets satisfying the VAF threshold were shortlisted from the total number of breakpoints (Supplementary Table 1). For conventional Ig/TCR MRD rearrangements in ALL, markers were detected by single or multiplex PCR, heteroduplex analysis and Sanger sequencing [15]. Assays with optimal performance as indicated by the correlation coefficient of DNA serial dilutions and PCR efficiency were selected as patient-specific targets and further evaluated as MRD markers.

### qPCR analysis of DNA markers

Patient-specific DNA qPCR assays were performed using a CFX96 Real-Time PCR Detection System (Bio-Rad). Standard curves using 10-fold dilution series of diagnostic DNA (500 ng) were prepared for each patient, according to Euro-MRD guidelines [18]. Primers and probes were designed with Primer Express 2.0.0 (Applied Biosystems) and Primer3 input software (version 4.1.0) and are listed in Supplementary Table 2. Each reaction was performed in 25 μL volume consisting of 12.5 μL of 2× iQ Supermix (Bio-Rad), 1.25 μL of 20× primers (500 nmol/L) and probe (200 nmol/L) and 500 ng of DNA (in 5 μL). Thermal cycling conditions consisted of pre-cycling hold for 10 min at 95 °C, ten touchdown cycles from 71 to 61 °C for 30 s at 95 °C, 40 cycles at 95 °C for 15 s and 61 °C for 1 min. All breakpoint assays were performed in triplicate along with PB DNA controls and a no-template control. Assays were optimised to reach high PCR efficiency (slope −3.1 to −3.9) and then tested on serially collected patient samples. MRD data were interpreted according to the standardised guidelines set by the Euro-MRD consortium [18].

### ddPCR for DNA markers

ddPCR was performed using a QX200 ddPCR system (Bio-Rad). The 20 μL of ddPCR reaction consisted of 10 μL of 2× ddPCR Master Mix for probes (Bio-Rad), 1 μL of 20× primers (500 nmol/L) and probe (200 nmol/L) and 5 μL of gDNA (100 ng/μL). Primers and probes used for ddPCR were used for qPCR assays. Droplets were generated by the QX200 droplet generator device and DNA was amplified with a C1000 Touch Thermal Cycler (Bio-Rad). Thermal cycling conditions were a pre-cycling hold at 95 °C for 10 min, ten touchdown cycles (71–61 °C) of 30 s at 94 °C, 40 cycles at 94 °C for 30 s and 61 °C for 1 min, and a post-cycling hold at 98 °C for 10 min. The ramp rate was 2 °C/s. After ddPCR amplification, the droplets were counted by qx200 Droplet Reader (Bio-Rad) and the raw data were analysed using the QuantaSoft Software version 1.7. Reactions with the number of accepted droplets <10,000 per well were excluded from the analysis. The DNA copy numbers in clinical samples were evaluated against the diagnostic samples for MRD assessment.

### qRT-PCR analysis of RNA-MRD markers

qRT-PCR analysis of NB mRNA transcripts and EWS mRNA fusion transcripts was performed following reverse transcription of the RNA samples using iScript^TM^ Advanced cDNA Conversion Kit (Bio-Rad). TaqMan RT-PCR assays were performed using the ABI PRISM^TM^ 7700 Sequence Detector (Applied Biosystems). The primers and probes used for RT-PCR analysis are listed in Supplementary Table 3. HR-NB and EWS mRNA transcripts were analysed as described previously [34, 35]. PCR was performed in 25 μL reactions consisting of 12.5 μL Taqman Universal PCR Master Mix (2×, Applied Biosystems), 0.25 μL of 10 μM forward and reverse primers (100 nM), 0.125 μL of 20 μM probe (100 nM) and 5 μL of complementary DNA generated from 100 ng of total RNA. Thermal cycling conditions were 50 °C for 2 min, 95 °C for 10 min, followed by 40 cycles at 95 °C for 15 s and 60 °C for 1 min. When no amplification occurs, due to the absence or no detectable presence of target mRNA, a *C*_t_ value of 40 was recorded. A comparative *C*_t_ method was used to analyse the mRNA expression normalised against *β2M* as the housekeeping gene.

### Analysis of discordances

Discordances in qPCR and ddPCR MRD assays were classified using established guidelines described previously [49]. Discordances were classified as major qualitative discordances when MRD detection was positive in only one of the assay methods. A quantitative discordance is recorded when the MRD values showed a >1 log discrepancy between qPCR and ddPCR detection methods. MRD values that were either non-quantitative or negative in both assay formats were not considered discordant.

### Statistics

Statistical tests and data analyses were performed using R and GraphPad Prism 8.0. Linear regression analysis was performed on standard curves generated from DNA dilutions. DNA-MRD data from qPCR and ddPCR experiments were analysed using Pearson’s correlation. Transcript level expression of RNA markers between two individual dilutions in spike-in experiments was analysed by Student’s *t* test. *P* < 0.05 was considered significant. For normality assumption in cases with data not showing normal distributions, a logarithmic transformation was performed.

## Results

### Patient and sample characteristics

Samples from 18 patients with high-risk cancer, enrolled on the TARGET or PRISM clinical trials, were analysed in this study. The cohort included patients diagnosed with relapsed ALL (*n* = 6), relapsed HR-NB (*n* = 6) and relapsed EWS (*n* = 6). The median ages at diagnosis for ALL, HR-NB and EWS were 7.9, 3.6 and 8.1 years, respectively. The patient characteristics are listed in Table 1. The status of Ig/TCR rearrangements, MYC amplification and EWS fusions are listed for ALL, HR-NB and EWS, respectively.

**Table 1:** Patient characteristics in ALL, HR-NB and EWS.

Patient ID	Age (yr)	Gender	Diagnosis	Status of *IG/TCR* rearrangement (ALL), *MYCN* (HR-NB), *EWSR*1 fusion (EWS)
ALL-1	9.9	F	T-ALL	*IGH: VH4-DH2-JH4*
ALL-2	2.1	M	B-ALL	*TCRB: DB1-JB1.5*
ALL-3	4.6	F	B-ALL	*TCRG: Vg3-Jg2*
ALL-4	14.7	F	B-ALL	*IGK: VK2-Kdel*
ALL-5	9.1	F	B-ALL	*TCRA: Vd2-Dd3-Ja29*
ALL-6	7.1	M	T-ALL	*TCRG: Vg11-Jg2*
HR-NB1	4.2	F	HR-NB	*MYCN* not amplified
HR-NB2	0.8	M	HR-NB	MYCN amplified
HR-NB3	2	F	HR-NB	*MYCN* not amplified
HR-NB4	5	M	HR-NB	*MYCN* not amplified
HR-NB5	7.5	M	HR-NB	*MYCN* not amplified
HR-NB6	3	F	HR-NB	*MYCN* not amplified
EWS-1	8	M	EWS	*EWSR1-FLI1*
EWS-2	1.2	M	EWS	*EWSR1-FLI1*
EWS-3	12	M	EWS	*EWSR1-FLI1*
EWS-4	8.3	F	EWS	*EWSR1-ETV1*
EWS-5	15.9	M	EWS	*EWSR1-ERG*
EWS-6	3.3	M	EWS	*EWSR1-ETV1*

*ALL* acute lymphoblastic leukaemia, *HR-NB* high-risk neuroblastoma, *EWS* Ewing sarcoma, *B-ALL* B acute lymphoblastic leukaemia, *T-ALL* T cell acute lymphoblastic leukaemia.

### Development of WGS-MRD assays for paediatric patients using qPCR

**Fig. 1:**
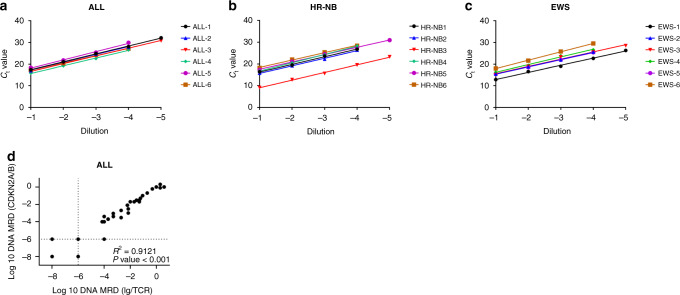
Analysis of WGS-MRD markers in ALL, HR-NB and EWS. Linear regression standard dilution curves for WGS-MRD markers detected by quantitative RT-PCR in **a** ALL, **b** HR-NB and **c** EWS. Slope and *R*^2^ values are shown in Table 2. *C*_t_ values represent the mean of triplicate experiments and are plotted against sample serial dilution based on the lowest quantitative range of 10^−4^ (0.01%) or 10^−5^ (0.001). **d** Pearson’s correlation analysis of patient-specific *CDKN2A/B* MRD assays to previously developed Ig/TCR assays using qPCR in clinical samples from six ALL patients. Each data point represents an individual clinical sample. Dotted lines indicate thresholds for positive, but non-quantitative (PNQ) MRD as assigned according to EuroMRD guidelines.

Patient-specific PCR assays were developed for each ALL, HR-NB and EWS patient using DNA breakpoints identified from WGS data. The sensitivity and quantitative range of qPCR assays were assessed by performing a series of 10-fold dilutions (10^−1^–10^−5^) of either patient tumour DNA or cognate PDX tumour DNA into DNA from control BM MNCs. The sensitivity and quantitative range of the assays were determined using the guidelines set by the Euro-MRD consortium [18]. The standard curves generated from the dilution series of ALL, HR-NB and EWS DNA represents the assay quantitative range (Fig. 1a–c and Supplementary Fig. 1a–c). All assays had a quantitative range of ≤10^−4^ (0.01%) and showed no amplification with genomic DNA obtained from normal PB cells. In ALL, HR-NB and EWS, 33% (2/6) of the tumours were quantitative down to ≤10^−5^ (0.001%). The assays showed excellent linearity (*R*^2^ > 0.98) and PCR efficiency (90–110%, slope value between −3.1 and –3.8) across all samples. Patient-specific breakpoints involving *CDKN2A/B* deletions were selected as WGS-MRD targets in ALL. WGS-MRD targets in HR-NB and EWS involved gene deletions, duplications and inversions in a patient-specific manner. In ALL, a single assay was designed and validated per patient. In HR-NB and EWS, multiple assays (up to 3) were designed per patient, and the ones that produced the highest linearity across sample dilutions were selected as MRD targets. WGS-MRD assays were successfully developed for every ALL, HR-NB and EWS patient, wherein ~90% of the assays tested were quantitative down to ≤10^−4^. The list of all assays tested by qPCR is provided in Supplementary Table 4. The chromosomal location, breakpoint and type of structural variation of the selected targets and the qPCR performance indicators in ALL, HR-NB and EWS patients are summarised in Table 2. Each ALL patient had existing Ig/TCR qPCR MRD assays, which had been previously used for MRD monitoring from their initial diagnosis. The assays were therefore directly compared against the patient-specific *CDKN2A/B* assays designed from WGS data (Fig. 1d). Analysis of MRD values obtained from clinical samples (*n* = 36) of six ALL patients revealed a significant correlation (*R*^2^ = 0.9121, *P* < 0.0001) between the Ig/TCR and patient-specific *CDKN2A/B* assays designed in our study. The MRD was determined as either positive, positive but not quantitative or negative according to the predefined criteria for MRD reporting in ALL [18]. A 94% (34/36) concordance was observed between the two assays, wherein two samples showed a >1 log discordance between the *CDKN2A/B* and *Ig/TCR* MRD assays.

**Table 2:** Summary of WGS-MRD qPCR assay performance in ALL, HR-NB and EWS.

Patient ID	WGS-MRD target gene	Chr	Breakpoint	Type	Slope value	*R*^2^ value	qPCR quantitative range	qPCR sensitivity
ALL-1	*CDKN2A/B*^*1*^	9	21901886	Del	−3.648	0.9993	10^−5^	10^−5^
ALL-2	*CDKN2A/B*^*2*^	9	21469564	Del	−3.584	0.9941	10^−4^	10^−5^
ALL-3	*CDKN2A/B*^*3*^	9	21948309	Del	−3.571	0.9995	10^−5^	10^−5^
ALL-4	*CDKN2A/B*^*4*^	9	21802667	Del	−3.555	0.9982	10^−4^	10^−4^
ALL-5	*CDKN2A/B*^*5*^	9	21975749	Del	−3.881	0.9952	10^−4^	10^−5^
ALL-6	*CDKN2A/B*^*6*^	9	21968001	Del	−3.516	0.9971	10^−4^	10^−5^
HR-NB1	*EZH2*	7	148541457	Del	−3.516	0.9971	10^−4^	10^−5^
HR-NB2	*TERT*	5	1296732	Dup	−3.472	0.9959	10^−4^	10^−4^
HR-NB3	*MYCN*	2	16013211	Dup	−3.509	0.9975	10^−5^	10^−5^
HR-NB4	*CNTN5*^*1*^	11	99492525	Del	−3.807	0.9995	10^−4^	10^−4^
HR-NB5	*CNTN5*^*2*^	11	99812931	Del	−3.363	0.9988	10^−5^	10^−5^
HR-NB6	*NDST4*	4	116044033	Del	−3.395	0.9974	10^−4^	10^−4^
EWS-1	*CDKN2A*	9	22035192	Del	−3.291	0.9959	10^−5^	10^−5^
EWS-2	*CCDC117*	22	29170280	Del	−3.363	0.9993	10^−4^	10^−5^
EWS-3	*PPP2R5*	14	10234781	Dup	−3.341	0.9867	10^−5^	10^−5^
EWS-4	*CPEB4*	5	173327179	Inv	−3.54	0.9927	10^−4^	10^−4^
EWS-5	*SnoU13*	1	231036549	Del	−3.311	0.9993	10^−4^	10^−4^
EWS-6	*RASGRF2*	5	80404590	Del	−3.875	0.999	10^−4^	10^−4^

*ALL* acute lymphoblastic leukaemia, *HR-NB* high-risk neuroblastoma, *EWS* Ewing sarcoma, *Chr* chromosome, *Amp* amplification, *Del* deletion, *Dup* duplication, *Inv* inversion.

CDKN2A/B^1–6^, CNTN5^1,2^: see Supplementary Table 1.

### Performance of WGS-MRD assays in ddPCR format

**Fig. 2:**
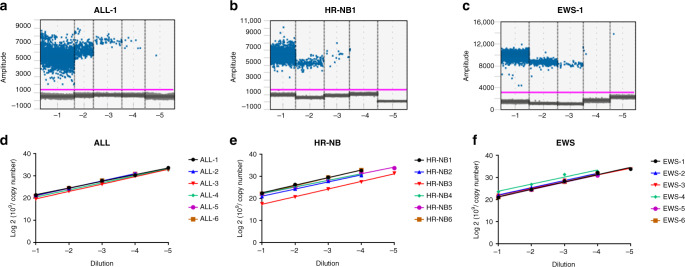
Representative 1D plots obtained by ddPCR analysis showing fluorescence produced by patient-specific probes and copy numbers in 10-fold serial dilutions of **a** ALL, **b** HR-NB and **c** EWS patient DNA. The FAM fluorescent signal (*y*-axis) of each droplet is plotted against the droplet cumulative count (*x*-axis). Each dot above the threshold line shows a positive droplet for the patient-specific DNA breakpoint target. **d**–**f** Linear regression standard curves obtained by log 2 transformation of ddPCR copy numbers in serial dilutions of ALL, HR-NB and EWS DNA.

Next, the validated WGS-MRD assays were assessed using ddPCR and the analytical performance was compared against qPCR format. The quantitative detection of patient-specific MRD targets in serial dilutions (10^−1^–10^−5^) of diagnostic DNA from each of the 18 ALL, HR-NB and EWS patients was determined. Representative one-dimensional (1D) plots of ddPCR reactions with positive droplets detected in DNA serial dilutions are shown in Fig. 2a–c. The positive droplets were converted to copy numbers and the ddPCR detected as little as one copy, or 10^−5^ pg of tumour DNA. The 1D plots and copy numbers in serial dilutions of all 18 patient samples are shown in Supplementary Fig. 2a–c. We fitted a linear regression model to the log-transformed copy number, showing high linearity of DNA copy numbers across sample dilutions (Fig. 2d–f and Supplementary Fig. 3a–c). Consistent with the qPCR data, quantitative detection of positive droplets was observed at dilutions ≤10^−4^ (0.01%) in all patient samples. An assay quantitation of ≤10^−5^ (0.001%) was observed in 33% (2/6) of ALL, HR-NB and EWS tumours (Fig. 2e, f). Similar to qPCR assays, the MRD ddPCR tests were highly specific with no background amplification detected for DNA from PB controls.

### WGS-MRD analysis of disease course and correlation of qPCR and ddPCR assays

**Fig. 3:**
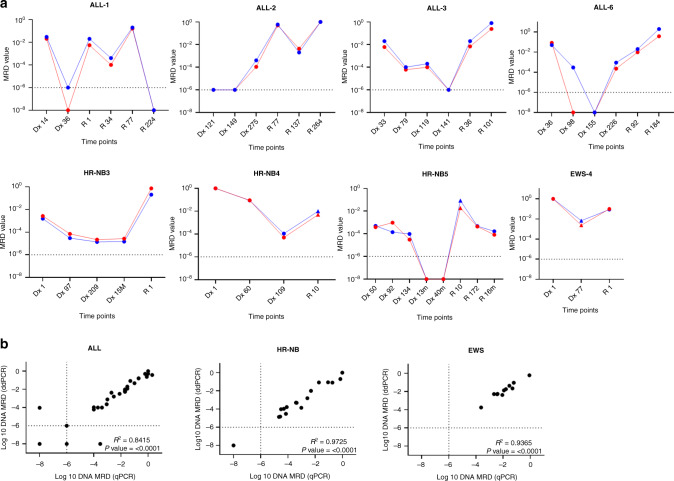
**a** WGS-MRD analysis of disease course by qPCR (red) and ddPCR (blue) in serially collected patient samples of ALL, HR-NB and EWS. Each panel represents an individual patient and MRD level detected at specific time points in clinical specimens of bone marrow (dot) or peripheral blood (triangle). ‘Dx’ and ‘R’ represents days/months (m) post-diagnosis and relapse, respectively. **b** Pearson’s correlation plots of MRD detected by qPCR and ddPCR in ALL, HR-NB and EWS. Each data point represents an individual clinical sample. Dotted lines indicate positive but non-quantitative (PNQ) MRD as assigned according to EuroMRD guidelines.

To assess the WGS-MRD assays in longitudinal experiments, we measured MRD in serially collected BM or PB for eight patients (four ALL, three HR-NB and one EWS) using both qPCR and ddPCR analysis (Fig. 3). MRD positivity was defined based on the assay sensitivity and quantitative range determined in qPCR assays using 10-fold dilutions of diagnostic DNA. The serial collections of patient samples with no detectable MRD were classified as MRD negative. The DNA assays were able to detect MRD in the PB and BM samples from diagnosis through relapse in ALL, HR-NB and EWS patients. The MRD values followed disease course and were comparable between qPCR and ddPCR platforms. In the majority of patients, a high MRD burden was detected at diagnosis and relapse time points. The proportion of tumour cells in MRD-positive samples ranged from ~0.001% (10^−5^) to ~100% (10^−0^) of MNCs. A total of 36 ALL, 18 HR-NB and 12 EWS samples were analysed by qPCR and ddPCR for assay concordance between the two platforms. A 100% assay concordance was observed in HR-NB (18/18) and EWS (12/12) samples, whereas ALL showed concordant results in 94% (34/36) samples using the pre-defined MRD positive thresholds. Correlation analysis of all the available clinical samples indicated a significant correlation between qPCR and ddPCR values (Fig. 3b); *R*^2^ = 0.84 for ALL, *R*^2^ = 0.97 for HR-NB and *R*^2^ = 0.93 for EWS (*P* < 0.0001 for each).

### Analysis of RNA-MRD and correlation with WGS-MRD in HR-NB and EWS

**Fig. 4:**
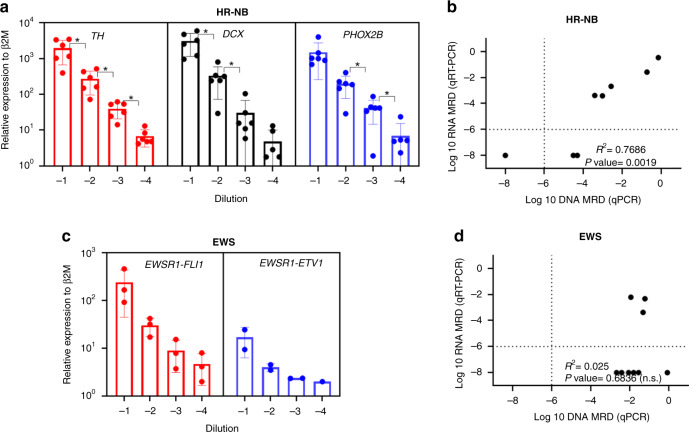
Relative expression of mRNA transcripts relative to β2M in healthy bone marrow (BM) cells spiked with **a** HR-NB and **b** EWS patient-derived xenograft cells. Cell spike-in ranges are 10,000 (10^−1^)–10 (10^−4^) HR-NB or EWS cells in bone marrow control cells. The MRD values were plotted against sample serial dilutions. Each dot represents average transcript levels in one patient from triplicate experiments. Error bars indicate mean ± standard deviation (SD). **c** and **d** Pearson’s correlation plots of DNA and RNA-based MRD analysis in HR-NB (**c**) and EWS (**d**) patient samples. *TH* expression in HR-NB and *EWSR1-FLI1* expression in EWS patients were compared against patient-specific WGS-MRD assays. Dotted lines indicate positive but non-quantitative (PNQ) MRD as assigned according to EuroMRD guidelines.

Finally, we evaluated the performance of the most common RNA-based MRD markers using qRT-PCR. Serial dilutions of HR-NB tumours (10^−1^–10^−4^) were prepared by diluting fixed numbers of tumour cells into control BM MNCs. Among the three NB mRNAs, *TH*, *PHOX2B* and *DCX*, *TH* mRNA showed a significant linear correlation (*R*^2^ = 0.9923, *P* = 0.0003) between the serial dilutions across all HR-NB sample dilutions (Fig. 4a and Supplementary Table 5a). Hence, *TH* mRNA levels were detected in clinical samples (*n* = 9) collected from six HR-NB patients and compared directly against patient-specific WGS-MRD assay. As shown in Fig. 4b, a significant correlation between the expression of *TH* mRNA transcripts and patient-specific DNA markers was observed in MRD-positive clinical specimens of HR-NB (*R*^2^ = 0.7686, *P* = 0.0019). Here, 78% (7/9) of samples showed a positive MRD burden by DNA breakpoint analysis, whereas RNA-MRD was positive only in 56% (5/9) samples. Similarly, serial dilutions of EWS tumours (10^−1^–10^−4^) were prepared by diluting fixed numbers of tumour cells into control BM MNCs and the mRNA expression of *EWSR1*-*FLI1* and *EWSR1*-*ETV1* was determined. The EWS fusions detected from WGS data are listed (Table 1). Consistently, qRT-PCR of EWS spike-in samples showed detection of *EWSR1-FLI1* fusion transcript in *n* = 3 and *EWSR1-ETV1* fusion in *n* = 2 patients (Fig. 4c). A high transcript-level expression and linear correlation were observed for *EWSR1-FLI1* fusion (*R*^2^ = 0.9998, *P* < 0.0001) as compared to *EWSR1-ETV1* fusion (*R*^2^ = 0.9781, *P* = 0.0014) across all dilutions. (Supplementary Table 5b). Further to this, *EWSR1-FLI1* mRNA was detected in clinical samples (*n* = 9) collected from three EWS patients and compared directly against patient-specific WGS-MRD assays. As shown in Fig. 4d, no significant correlation was observed between *EWSR1-FLI1* mRNA detection and patient-specific DNA markers. Of interest, patient-specific DNA-MRD analysis revealed a positive MRD detection in 100% (9/9) of EWS clinical samples, whereas RNA-MRD analysis of EWS fusion was positive only in 33% (3/9) of samples.

## Discussion

Tumour-specific genetic alterations are ideal candidates for monitoring therapeutic response, disease progression and early detection of relapse. The limited availability of patient- and tumour-specific WGS data has, up until recently, been a significant limiting factor. In this study, we utilised WGS data from the TARGET and PRISM personalised medicine clinical trials [45] to identify patient- and tumour-specific MRD assays and to highlight that the clinical utility of patient-specific WGS data extends beyond the identification of therapeutically targetable genomic lesions. With wider availability of WGS analysis of tumours at diagnosis, WGS data will facilitate the development of patient-specific MRD assays, as well as identifying diagnostic molecular lesions (e.g. *EWSR1* translocations in EWS), stratifying molecular features (e.g. *MYCN* amplification in HR-NB) and identifying targetable lesions (e.g. *ALK* mutation in HR-NB). Here, we show that DNA breakpoints identified by WGS of the primary tumour can reliably be used as PCR-based patient-specific MRD markers in three paediatric cancers, ALL, HR-NB and EWS. The patient-specific DNA markers were stable through the course of treatment and were able to track the disease course. In the PRISM study, the average turnaround time from patient enrolment to return of data to the molecular tumour board was 7.5 weeks [45]. A validated MRD assay can be developed and applied within 6 weeks of receiving the genomic data, making it feasible to implement real-time MRD measurements from mid-induction induction therapy onwards for patients with HR-NB and EWS.

Unlike adult tumours, paediatric cancers have a significantly lower mutation rate but harbour a large number of structural chromosomal abnormalities [50, 51]. Targeting these DNA breakpoints facilitates the design of patient-specific assays that directly relates to the oncogenic process. ALL was the first neoplasm where the assessment of therapeutic response by MRD monitoring has proven to be a fundamental tool for guiding treatment decisions [1–16]. In contrast, patient-specific MRD assays in solid paediatric tumours are underdeveloped and current approaches rely largely on measuring the expression of tumour-associated genes or fusions. This study shows that it is feasible for solid paediatric cancers to adopt and implement the quality assurance and control guidelines for DNA-based MRD detection developed for ALL for multi-laboratory standardisation by the Euro-MRD consortium [17–19, 52] and which have been developed for mRNA-based MRD in NB [34, 53]. This provides a rationale for prospectively comparing multiple methods of MRD detection including current RNA-based approaches [43], hypermethylated *RASSF1* [38, 54], miRNA [39], cfDNA [40, 41] and multiplexed RNA [42], as well as DNA-based qPCR. One potential advantage of PCR-based MRD testing is the capacity for accurate quantitation of tumour cell burden, allowing for the detection of 1 tumour cell in 10,000 (10^−4^) to 100,000 (10^−5^) normal cells. Although qPCR analysis of Ig/TCR gene rearrangements is a common tool for MRD detection in ALL, here we used assays targeting patient-specific breakpoints of *CDKN2A/B* deletions to confirm the MRD targets identified by WGS are equivalent to conventional Ig/TCR targets. Since *CDNK2A/B* deletions are the most common microdeletion in ALL and present in both T and B-ALL patients [55], these markers could potentially overcome the occasional lack of suitable *Ig/TCR* markers in ALL patients [19, 56].

This study utilised PDX-derived DNA for the development of patient-specific assays in HR-NB and EWS. Previously, we have shown that tumours formed in PDX models of NB recapitulate the patient tumour characteristics both genetically and histologically [46]. This is consistent with other studies that have shown a close resemblance of PDX tumours with that of the parental tumours in multiple cancers [46, 57]. Although WGS initially identified multiple DNA breakpoints for each patient, the ones with the highest VAF used in this study served as ideal assay targets that correlated with the disease course during treatment. More importantly, the MRD assays designed using diagnostic tumour DNA were able to detect disseminated disease in both PB and BM and provided a retrospective estimate of disease burden. We have not undertaken a systematic comparison of MRD levels in matched PB and BM at the same time points in this cohort, but note that high levels of MRD were detectable in either PB or BM at different times in all four patients with HR-NB or EWS.

All the MRD assays designed to target DNA breakpoints showed high concordance between qPCR and ddPCR methods. Moreover, when tested on primary samples, ddPCR displayed significant concordance with qPCR in all tumour types. Although this study was conducted in a small cohort of patients, our findings nevertheless provide substantive evidence towards the clinical utility of DNA-based MRD detection for therapy monitoring in solid paediatric cancers. Our findings add credence to the previous demonstration of sensitive DNA-based MRD detection in HR-NB [58] and further provides a uniform framework for assay development and feasibility across multiple cancers. In solid paediatric tumours, DNA-MRD represents a promising alternative to current RNA-based approaches primarily because this method is independent of the heterogeneity in gene expression levels between patients. Previously, RT-qPCR for NB-specific target genes, including *TH*, *DCX* and *PHOX2B*, was shown to be useful for MRD detection in the BM and PB samples of HR-NB [43, 53]. However, these assays often show non-specific expression in normal cells of the hematopoietic lineage, and MRD detection is based on the cut-off value between the expression levels in NB cells from normal cells [34, 59].

This study adopted the standardised operating procedures for MRD detection by RT-qPCR as defined by the European Society of Pediatric Oncology Neuroblastoma Group (SIOPEN) [34]. Among the three NB-specific mRNAs tested in this study, *TH* expression was detected consistently across all the dilutions in our in vitro spike-in model and in clinical samples of HR-NB. Although the three mRNAs revealed a detection sensitivity up to 10^−4^ (0.01%), *DCX* and *PHOX2B* were not detected in the lowest dilution in 1/6 HR-NB patients. The detection of *EWSR1* fusion transcript expression by RT-qPCR is a common method for MRD detection in EWS tumours. The EWS tumours analysed in this study had *EWS* translocations with *FLI1* (*n* = 3) and *ETV1* (*n* = 2). Analysis of these fusion transcripts showed a detection sensitivity up to 10^−4^ (0.01%) as shown by the in vitro spike-in experiments with BM controls. Here, analysis of *TH* and *EWSR1-FLI1* transcripts in clinical specimens of BM and PB revealed RNA-MRD assays as less sensitive upon comparison with DNA-MRD assays. RNA-MRD is likely to be more prone to errors as a result of tumour heterogeneity and the low stability of RNA molecules. A significant correlation between DNA and RNA-MRD was observed in HR-NB; however, no such correlation was observed in EWS patients.

Among the paediatric solid tumours, HR-NB and EWS are highly aggressive in nature with early metastasis and relapse occurring at multiple anatomical sites. WGS of primary and metastatic lesions in these tumours may facilitate the identification of DNA aberrations, which are unique to the disseminated site and provide a deeper insight into disease progression and prognosis [60, 61]. Whilst patient-specific, DNA-based MRD detection is feasible, its clinical implications need further investigation in the context of prospective clinical trials.

In summary, this study confirms that WGS data can be used to identify individualised, tumour-specific DNA breakpoints and can be used as reliable targets for MRD analysis in paediatric cancers. Patient-specific DNA-MRD analysis should be prospectively tested alongside other approaches including RNA-MRD [43], miRNA [39] and methylated *RASSF1* [38] to determine their clinical utility for monitoring treatment response and disease progression. Furthermore, this study shows that a framework for the quantitative analysis and interpretation of solid tumour MRD based on the Euro-MRD guidelines [18, 19] was effective and has potential applicability across multiple cancers and treatment protocols.

## Supplementary information

Supplemental Tables & Supplementary Figure Legends

Supplemental Figure 1

Supplemental Figure 2

Supplemental Figure 3

Reproducibility checklist

## References

[1] Conter V, Bartram CR, Valsecchi MG, Schrauder A, Panzer-Grümayer R, Möricke A (2010). Molecular response to treatment redefines all prognostic factors in children and adolescents with B-cell precursor acute lymphoblastic leukemia: results in 3184 patients of the AIEOP-BFM ALL 2000 study. Blood.

[2] Schrappe M, Valsecchi MG, Bartram CR, Schrauder A, Panzer-Grümayer R, Möricke A (2011). Late MRD response determines relapse risk overall and in subsets of childhood T-cell ALL: results of the AIEOP-BFM-ALL 2000 study. Blood.

[3] Vora A, Goulden N, Wade R, Mitchell C, Hancock J, Hough R (2013). Treatment reduction for children and young adults with low-risk acute lymphoblastic leukaemia defined by minimal residual disease (UKALL 2003): a randomised controlled trial. Lancet Oncol.

[4] Vora A, Goulden N, Mitchell C, Hancock J, Hough R, Rowntree C (2014). Augmented post-remission therapy for a minimal residual disease-defined high-risk subgroup of children and young people with clinical standard-risk and intermediate-risk acute lymphoblastic leukaemia (UKALL 2003): a randomised controlled trial. Lancet Oncol.

[5] Pui CH, Pei D, Coustan-Smith E, Jeha S, Cheng C, Bowman WP (2015). Clinical utility of sequential minimal residual disease measurements in the context of risk-based therapy in childhood acute lymphoblastic leukaemia: a prospective study. Lancet Oncol.

[6] Borowitz MJ, Wood BL, Devidas M, Loh ML, Raetz EA, Salzer WL (2015). Prognostic significance of minimal residual disease in high risk B-ALL: a report from Children’s Oncology Group study AALL0232. Blood.

[7] Bartram J, Wade R, Vora A, Hancock J, Mitchell C, Kinsey S (2016). Excellent outcome of minimal residual disease-defined low-risk patients is sustained with more than 10 years follow-up: results of UK paediatric acute lymphoblastic leukaemia trials 1997-2003. Arch Dis Child.

[8] Parker C, Waters R, Leighton C, Hancock J, Sutton R, Moorman AV (2010). Effect of mitoxantrone on outcome of children with first relapse of acute lymphoblastic leukaemia (ALL R3): an open-label randomised trial. Lancet.

[9] Bader P, Kreyenberg H, von Stackelberg A, Eckert C, Salzmann-Manrique E, Meisel R (2015). Monitoring of minimal residual disease after allogeneic stem-cell transplantation in relapsed childhood acute lymphoblastic leukemia allows for the identification of impending relapse: results of the ALL-BFM-SCT 2003 trial. J Clin Oncol.

[10] Marshall GM, Dalla Pozza L, Sutton R, Ng A, de Groot-Kruseman HA, van der Velden VH (2013). High-risk childhood acute lymphoblastic leukemia in first remission treated with novel intensive chemotherapy and allogeneic transplantation. Leukemia.

[11] Eckert C, Henze G, Seeger K, Hagedorn N, Mann G, Panzer-Grümayer R (2013). Use of allogeneic hematopoietic stem-cell transplantation based on minimal residual disease response improves outcomes for children with relapsed acute lymphoblastic leukemia in the intermediate-risk group. J Clin Oncol.

[12] Bader P, Salzmann-Manrique E, Balduzzi A, Dalle JH, Woolfrey AE, Bar M (2019). More precisely defining risk peri-HCT in pediatric ALL: pre- vs post-MRD measures, serial positivity, and risk modeling. Blood Adv.

[13] Karsa M, Dalla Pozza L, Venn NC, Law T, Shi R, Giles JE (2013). Improving the identification of high risk precursor B acute lymphoblastic leukemia patients with earlier quantification of minimal residual disease. PLoS ONE.

[14] Pulsipher MA, Carlson C, Langholz B, Wall DA, Schultz KR, Bunin N (2015). IgH-V(D)J NGS-MRD measurement pre- and early post-allotransplant defines very low- and very high-risk ALL patients. Blood.

[15] Sutton R, Shaw PJ, Venn NC, Law T, Dissanayake A, Kilo T (2015). Persistent MRD before and after allogeneic BMT predicts relapse in children with acute lymphoblastic leukaemia. Br J Haematol.

[16] Della Starza I, Chiaretti S, De Propris MS, Elia L, Cavalli M, De Novi LA (2019). Minimal residual disease in acute lymphoblastic leukemia: technical and clinical advances. Front Oncol.

[17] van Dongen JJ, Langerak AW, Brüggemann M, Evans PA, Hummel M, Lavender FL (2003). Design and standardization of PCR primers and protocols for detection of clonal immunoglobulin and T-cell receptor gene recombinations in suspect lymphoproliferations: report of the BIOMED-2 Concerted Action BMH4-CT98-3936. Leukemia.

[18] van der Velden VH, Cazzaniga G, Schrauder A, Hancock J, Bader P, Panzer-Grumayer ER (2007). Analysis of minimal residual disease by Ig/TCR gene rearrangements: guidelines for interpretation of real-time quantitative PCR data. Leukemia.

[19] van Dongen JJ, van der Velden VH, Brüggemann M, Orfao A (2015). Minimal residual disease diagnostics in acute lymphoblastic leukemia: need for sensitive, fast, and standardized technologies. Blood.

[20] Theunissen P, Mejstrikova E, Sedek L, van der Sluijs-Gelling AJ, Gaipa G, Bartels M (2017). Standardized flow cytometry for highly sensitive MRD measurements in B-cell acute lymphoblastic leukemia. Blood.

[21] Schumich A, Maurer-Granofszky M, Attarbaschi A, Pötschger U, Buldini B, Gaipa G (2019). Flow-cytometric minimal residual disease monitoring in blood predicts relapse risk in pediatric B-cell precursor acute lymphoblastic leukemia in trial AIEOP-BFM-ALL 2000. Pediatr Blood Cancer.

[22] Venn NC, van der Velden VH, de Bie M, Waanders E, Giles JE, Law T (2012). Highly sensitive MRD tests for ALL based on the IKZF1 Δ3-6 microdeletion. Leukemia.

[23] Uemura S, Ishida T, Thwin KKM, Yamamoto N, Tamura A, Kishimoto K (2019). Dynamics of minimal residual disease in neuroblastoma patients. Front Oncol.

[24] Wagner LM, Smolarek TA, Sumegi J, Marmer D (2012). Assessment of minimal residual disease in ewing sarcoma. Sarcoma.

[25] Cotterill SJ, Pearson AD, Pritchard J, Foot AB, Roald B, Kohler JA (2000). Clinical prognostic factors in 1277 patients with neuroblastoma: results of the European Neuroblastoma Study Group ‘Survey’ 1982-1992. Eur J Cancer.

[26] Pinto NR, Applebaum MA, Volchenboum SL, Matthay KK, London WB, Ambros PF (2015). Advances in risk classification and treatment strategies for neuroblastoma. J Clin Oncol.

[27] Méhes G, Luegmayr A, Kornmüller R, Ambros IM, Ladenstein R, Gadner H (2003). Detection of disseminated tumor cells in neuroblastoma: 3 log improvement in sensitivity by automatic immunofluorescence plus FISH (AIPF) analysis compared with classical bone marrow cytology. Am J Pathol.

[28] Burchill SA, Beiske K, Shimada H, Ambros PF, Seeger R, Tytgat GA (2017). Recommendations for the standardization of bone marrow disease assessment and reporting in children with neuroblastoma on behalf of the International Neuroblastoma Response Criteria Bone Marrow Working Group. Cancer.

[29] Campbell KM, Shulman DS, Grier HE, DuBois SG (2021). Role of bone marrow biopsy for staging new patients with Ewing sarcoma: a systematic review. Pediatr Blood Cancer.

[30] Schleiermacher G, Peter M, Oberlin O, Philip T, Rubie H, Mechinaud F (2003). Increased risk of systemic relapses associated with bone marrow micrometastasis and circulating tumor cells in localized ewing tumor. J Clin Oncol.

[31] London WB, Castel V, Monclair T, Ambros PF, Pearson AD, Cohn SL (2011). Clinical and biologic features predictive of survival after relapse of neuroblastoma: a report from the International Neuroblastoma Risk Group project. J Clin Oncol.

[32] Barker LM, Pendergrass TW, Sanders JE, Hawkins DS (2005). Survival after recurrence of Ewing’s sarcoma family of tumors. J Clin Oncol.

[33] Stutterheim J, Gerritsen A, Zappeij-Kannegieter L, Yalcin B, Dee R, van Noesel MM (2009). Detecting minimal residual disease in neuroblastoma: the superiority of a panel of real-time quantitative PCR markers. Clin Chem.

[34] Viprey VF, Corrias MV, Kagedal B, Oltra S, Swerts K, Vicha A (2007). Standardisation of operating procedures for the detection of minimal disease by QRT-PCR in children with neuroblastoma: quality assurance on behalf of SIOPEN-R-NET. Eur J Cancer.

[35] Chaput L, Grèze V, Halle P, Radosevic-Robin N, Pereira B, Véronèse L, et al. Sensitive and specific detection of Ewing sarcoma minimal residual disease in ovarian and testicular tissues in an in vitro model. Cancers. 2019;11:1807. 10.3390/cancers11111807. 10.3390/cancers11111807 PMC6895895 31744224

[36] Bridge RS, Rajaram V, Dehner LP, Pfeifer JD, Perry A (2006). Molecular diagnosis of Ewing sarcoma/primitive neuroectodermal tumor in routinely processed tissue: a comparison of two FISH strategies and RT-PCR in malignant round cell tumors. Mod Pathol.

[37] Stutterheim J, Zappeij-Kannegieter L, Ora I, van Sluis PG, Bras J, den Ouden E (2012). Stability of PCR targets for monitoring minimal residual disease in neuroblastoma. J Mol Diagn.

[38] van Zogchel LMJ, van Wezel EM, van Wijk J, Stutterheim J, Bruins WSC, Zappeij-Kannegieter L, et al. Hypermethylated RASSF1A as circulating tumor DNA marker for disease monitoring in neuroblastoma. JCO Precis Oncol. 2020;4:PO.19.00261. 10.1200/PO.19.00261. 10.1200/PO.19.00261 PMC7446415 32923888

[39] Morini M, Cangelosi D, Segalerba D, Marimpietri D, Raggi F, Castellano A, et al. Exosomal microRNAs from longitudinal liquid biopsies for the prediction of response to induction chemotherapy in high-risk neuroblastoma patients: a proof of concept SIOPEN study. Cancers. 2019;11:1476. 10.3390/cancers11101476. 10.3390/cancers11101476 PMC6826693 31575060

[40] Peitz C, Sprüssel A, Linke RB, Astrahantseff K, Grimaldi M, Schmelz K, et al. Multiplexed quantification of four neuroblastoma DNA targets in a single droplet digital PCR reaction. J Mol Diagn. 2020. 10.1016/j.jmoldx.2020.07.006. 10.1016/j.jmoldx.2020.07.006 32858250

[41] Peneder P, Stütz AM, Surdez D, Krumbholz M, Semper S, Chicard M (2021). Multimodal analysis of cell-free DNA whole-genome sequencing for pediatric cancers with low mutational burden. Nat Commun.

[42] van Zogchel LMJ, Zappeij-Kannegieter L, Javadi A, Lugtigheid M, Gelineau NU, Lak NSM, et al. Specific and sensitive detection of neuroblastoma mRNA markers by multiplex RT-qPCR. Cancers. 2021;13:150. 10.3390/cancers13010150. 10.3390/cancers13010150 PMC7796198 33466359

[43] Viprey VF, Gregory WM, Corrias MV, Tchirkov A, Swerts K, Vicha A (2014). Neuroblastoma mRNAs predict outcome in children with stage 4 neuroblastoma: a European HR-NBL1/SIOPEN study. J Clin Oncol.

[44] Gaspar N, Hawkins DS, Dirksen U, Lewis IJ, Ferrari S, Le Deley MC (2015). Ewing sarcoma: current management and future approaches through collaboration. J Clin Oncol.

[45] Wong M, Mayoh C, Lau LMS, Khuong-Quang DA, Pinese M, Kumar A (2020). Whole genome, transcriptome and methylome profiling enhances actionable target discovery in high-risk pediatric cancer. Nat Med.

[46] Kamili A, Gifford AJ, Li N, Mayoh C, Chow SO, Failes TW (2020). Accelerating development of high-risk neuroblastoma patient-derived xenograft models for preclinical testing and personalised therapy. Br J Cancer.

[47] Tsai CH, Tang JL, Tien FM, Kuo YY, Wu DC, Lin CC (2021). Clinical implications of sequential MRD monitoring by NGS at 2 time points after chemotherapy in patients with AML. Blood Adv.

[48] Kim T, Moon JH, Ahn JS, Kim YK, Lee SS, Ahn SY (2018). Next-generation sequencing-based posttransplant monitoring of acute myeloid leukemia identifies patients at high risk of relapse. Blood.

[49] Ladetto M, Brüggemann M, Monitillo L, Ferrero S, Pepin F, Drandi D (2014). Next-generation sequencing and real-time quantitative PCR for minimal residual disease detection in B-cell disorders. Leukemia.

[50] Stiller CA (2004). Epidemiology and genetics of childhood cancer. Oncogene.

[51] Vogelstein B, Papadopoulos N, Velculescu VE, Zhou S, Diaz LA, Kinzler KW (2013). Cancer genome landscapes. Science.

[52] van der Velden VH, Panzer-Grümayer ER, Cazzaniga G, Flohr T, Sutton R, Schrauder A (2007). Optimization of PCR-based minimal residual disease diagnostics for childhood acute lymphoblastic leukemia in a multi-center setting. Leukemia.

[53] Viprey VF, Lastowska MA, Corrias MV, Swerts K, Jackson MS, Burchill SA (2008). Minimal disease monitoring by QRT-PCR: guidelines for identification and systematic validation of molecular markers prior to evaluation in prospective clinical trials. J Pathol.

[54] Stutterheim J, Ichou FA, den Ouden E, Versteeg R, Caron HN, Tytgat GA (2012). Methylated RASSF1a is the first specific DNA marker for minimal residual disease testing in neuroblastoma. Clin Cancer Res.

[55] Sutton R, Venn NC, Law T, Boer JM, Trahair TN, Ng A (2018). A risk score including microdeletions improves relapse prediction for standard and medium risk precursor B-cell acute lymphoblastic leukaemia in children. Br J Haematol.

[56] Brüggemann M, Kotrova M (2017). Minimal residual disease in adult ALL: technical aspects and implications for correct clinical interpretation. Blood Adv.

[57] Zhang X, Claerhout S, Prat A, Dobrolecki LE, Petrovic I, Lai Q (2013). A renewable tissue resource of phenotypically stable, biologically and ethnically diverse, patient-derived human breast cancer xenograft models. Cancer Res.

[58] van Wezel EM, Zwijnenburg D, Zappeij-Kannegieter L, Bus E, van Noesel MM, Molenaar JJ (2015). Whole-genome sequencing identifies patient-specific DNA minimal residual disease markers in neuroblastoma. J Mol Diagn.

[59] Thwin KKM, Ishida T, Uemura S, Yamamoto N, Lin KS, Tamura A (2020). Level of seven neuroblastoma-associated mRNAs detected by droplet digital PCR is associated with tumor relapse/regrowth of high-risk neuroblastoma patients. J Mol Diagn.

[60] Abbasi MR, Rifatbegovic F, Brunner C, Mann G, Ziegler A, Pötschger U (2017). Impact of disseminated neuroblastoma cells on the identification of the relapse-seeding clone. Clin Cancer Res.

[61] Chicard M, Colmet-Daage L, Clement N, Danzon A, Bohec M, Bernard V (2018). Whole-exome sequencing of cell-free DNA reveals temporo-spatial heterogeneity and identifies treatment-resistant clones in neuroblastoma. Clin Cancer Res.

