# Supplementary material for: Whole-genome sequencing facilitates patient-specific quantitative PCR-based minimal residual disease monitoring in acute lymphoblastic leukaemia, neuroblastoma and Ewing sarcoma
Source: Br J Cancer. 2021 Sep 1;126(3):482–91. doi: 10.1038/s41416-021-01538-z (PMC8810788; doi:10.1038/s41416-021-01538-z)
Supplement: Supplementary file 1 — Supplemental Tables & Supplementary Figure Legends [file 41416_2021_1538_MOESM1_ESM.docx]

**Supplementary data**

**Supplementary Tables**

**Table 1.** Number of WGS breakpoints identified per patient in HR-NB and EWS

**Table 2.** List of DNA-MRD primers and probes in ALL, HR-NB and EWS

**Table 3.** List of RNA-MRD primers and probes in HR-NB and EWS

**Table 4.** List of all WGS-MRD targets, VAF’s and qPCR assay performance in HR-NB and EWS

**Table 5.** Linear regression analysis of HR-NB and EWS specific mRNA in bone marrow spike-in experiments. (a) Mean expression values of *TH*, *PHOX2B* and *DCX* mRNA transcripts from HR-NB patients (n=6) plotted against the respective spike-in dilutions. (b) Mean expression values of *EWS-FLI* and *EWS-ETV1* mRNA from patient tumors (n=3 and n=2, respectively) were plotted against the serial dilutions of bone marrow spike-ins.

**Supplementary Figures**

**Figure 1.** Individual linear regression standard curves of DNA break-point targets analyzed by qPCR in ten-fold serial dilutions of (a) ALL (b) HR-NB and (c) EWS patient DNA.

**Figure 2.** 1-D plots obtained by ddPCR analysis showing fluorescence produced by patient specific probes and copy numbers in 10-fold serial dilutions of (a) ALL (b) HR-NB and (c) EWS patient DNA. (d-f) Concentration plots of DNA dilutions obtained by QuantaSoft Analysis software. The average copies of target per droplet is calculated by applying a Poisson distribution to the number of positive droplets divided by total accepted droplets. Concentrations (copies/μL) represent the measurement of tumour DNA dilution in ‘merged’ wells for each sample. Error bars indicate the Poisson 95% confidence intervals for each measurement.

**Figure 3.**  Individual linear regression standard curves of DNA break-point targets obtained by Log2 transformation of ddPCR copy numbers in serial dilutions of (a) ALL (b) HR-NB and (c) EWS DNA.

**Table 1.** Number of WGS breakpoints identified per patient in HR-NB and EWS

| **Patient ID** | **Total number of breakpoints** | | **Breakpoints with VAF ≥ 0.3** | |
| --- | --- | --- | --- | --- |
| HR-NB1  HR-NB2  HR-NB3  HR-NB4  HR-NB5  HR-NB6  EWS-1  EWS-2  EWS-3  EWS-4  EWS-5  EWS-6 | 75  93  1230  547  218  21  170  350  110  411  683  24 | 58  30  62  100  111  10  35  47  42  14  115  2 | |  |

**Table 2.** List of WGS-MRD primers and probes in ALL, HR-NB and EWS.

| **Gene** | **F. Primer (5’-3’)** | **R. Primer (5’-3’)** | **Probe (5’FAM-3’IB)** |
| --- | --- | --- | --- |
| **ALL** | | | |
| *CDKN2A^1^* | TCTCACCATTCACAATGTAGAGAAACA | CAAAGAGTTTCTTTTGAAGCGAGG | ACATAGCTACCTACTACTGT |
| *CDKN2A^2^* | CCCTGGTTTCAAAGACAATTTCC | AATTCAGGATCCCCCAAAGG | AGGTGACATTGATATTGCAAAGAACCTGGC |
| *CDKN2A^3^* | GGGAAGCGGGAGTAATATTCG | CCCCAACCGCCTCCTTA | TTGGAGAAGGCTCTAGGCTGACCGTA |
| *CDKN2A^4^* | GGTGGGAGCTACGGGAATTTT | CAGAGTTAAAGCAGGAGAGAGGTTGT | CCCTGGTCACCGTCTCCTCAGGTG |
| *CDKN2A^5^* | GGACAGAATTCACTCTCACCATCA | CTGCCACCCTAGGGCGA | AGCCTGCAGCCTGAA |
| *CDKN2A^6^* | CCCTGGTTTCAAAGACAATTTCC | CATATCCCCCAGTCACGCTC | AGGTGACATTGATATTGCAAAGAACCTGGC |
| *IGH* | GTATTACTGTGCGAGACGGGATT | ACAACCTCTCTCCTGCTTTAACTCTG | CCCTGGTCACCGTCTCCTCAGGTG |
| *TCRB* | TGTACAAAGCTGTAACACCGTCTCAG | CCCTGATTCTGCAACTTACCTAGGAT | CAGCATTTTGGTGATGGGACTCGACTCTC |
| *TCRG* | CTGCTGGATTAGGCACTACTACCCTAT | AAGGTAATAGAGGGAAGGCAGGAA | TGTCACAGGTAAGTATCGGAAGAATACAACATTTCC |
| *IGK* | CCCTGGTTTCAAAGACAATTTCC | GGCCCAGTTGGGGTTGTG | AGGTGACATTGATATTGCAAAGAACCTGGC |
| *TCRA* | CTCTACAAACTCGCCCCGTG | CCTCCCTAGGAAGCCTCCCT | AGCTGCATTTTTGCCATATCCACTATTTGGAG |
| *TCRG* | CCAGGAAAGTATTATACTCATACACCCAG | TCCACTGCCCGGAGGAC | AGGTGGAGCTGGATATTG |
| **HR-NB** | | | |
| *EZH2* | TCCTAGCACTTTGGGAGACC | CCTTTCCGCCTTTTTAAACC | CCTGGGCAGCATAATGAGACACAAC |
| *MYC* | AGCGGGACAGAATAGACTGAA | TTCCTGCGACAAATATGTGAGT | CCTGAGTCCTGAATGGTCTTCCACTT |
| *TERT* | GGGAAAGTTAAAGGGAGTATTGAA | CACCCATAATACTGGGGTGTC | CCTGTAGAAGTAGGTAACGCCTCCCG |
| *CNTN5^1^* | GGGGCAACTCAAGATAGCTC | AATGACTGCATATAAACATCAGTGTA | TATGCCACGTTTGCATACATTTCTCAA |
| *CNTN5^2^* | TTTTGAGACTTCTCCAATTATAAAACA | CATGCTATAGAACCATGCCATATA | AAATCCCAGCACTCTTATATTTGCCCACA |
| *NDST4* | AGTGAGAGACCCAGGGTGAG | TCCTGTTTAATTGGAAATTTGG | GTGGGGGAGGACAGTAAAAA |
| **EWS** |  |  |  |
| *CDKN2A* | CCAAGGACAATGGAGGAAAT | ATTTCAGTGATGAACCAGCAA | AAATAAGTGCTGCTGAGGGC |
| *CCDC117* | GGCTTCATGCCATTCTCC | AGAGGAAGCAAGAGCTGTGAT | GAGACGGGGTTTCACTGTGT |
| *PPP2R5* | GAGTCGAGTTCGCACCACTA | CATTACTGTTTCCATGTCATACATTT | GGGAGACGTCACCTCAAAAA |
| *CPEB4* | GTCAGGCTGGTCTCGAACTC | TCTTTGGACATCACCAGGAA | GGCCTGGGTATGGTATCCTT |
| *SnoU13* | CAAAGCTGACTCCAGAGCAA | TACCAGCACCCCAGAATGTA | TCGAGAGCTACTGTAGTCTGCTG |
| *RASGRF2* | TGGTCAGAATTCTTGGGAAA | CTGCAATCCACTGCTTTTGT | CATTCAGGGGAAGTACAGCTG |

**Table 3.** List of RNA-MRD primers and probes in HRNB and EWS

| **Gene** | **F. Primer (5’-3’)** | **R. Primer (5’-3’)** | **Probe (5’FAM-3’TAMRA)** |
| --- | --- | --- | --- |
| **HR-NB** | | | |
| *TH* | ATTGCTGAGATCGCCTTCCA | AATCTCCTCGGCGGTGTACTC | ACAGGCACGGCGACCCGATTC |
| *PHOX2B* | CAGGGACCACCAGAGCAGT | CTGCTTGCGCTTCTCGTTGA | TACGCCGCAGTTCCTTACAAACTCTTCAC |
| *DCX* | CGCTATGCTCAGGATGATTTTTC | GCTGTGGCTGATGGGTTTCC | CATGACTCGGCATTCATTTTCATCCAG |
| **EWS** | | | |
| *EWSR1-FLI1* | CCAAGTCAATATAGCCAACAG | GGCCAGAATTCATGTTATTGC | ACGGGCAGCAGAACCCTTCTTATCACA |
| *EWSR1-ETV1* | CTGTCGGAGAGCAGCTCCAG | TAAATTCCATGCCTCGACCAG | CTCCTACCAGCTATTCCTCTACACAGCCGACT |
| *β2M* | GAGTATGCCTGCCGTGTG | AATCCAAATGCGGCATCT | CCTCCATGATGCTGCTTACATGTCTC |

**Table 4.** List of all WGS-MRD targets, variant allele frequencies and qPCR assay performance in HR-NB and EWS.

| **WGS-MRD targets** | **VAF** | **PCR**  **Quantitative range** | | **PCR Sensitivity** |
| --- | --- | --- | --- | --- |
| HR-NB1 |  |  | |  |
| EZH2 Del | 0.51 | 10^-4^ | | 10^-5^ |
| CDH22 | 0.78 | 10^-4^ | | 10^-4^ |
| HR-NB2 |  |  | |  |
| TERT Dup | 0.372 | 10^-4^ | | 10^-4^ |
| BCL10 Del | 0.346 | 10^-3^ | | 10^-4^ |
| HR-NB3 |  |  | |  |
| MYC Dup | 0.987 | 10^-5^ | | 10^-5^ |
| ALK Dup | 0.436 | 10^-4^ | | 10^-4^ |
| MYT1 Del | 0.3 | 10^-4^ | | 10^-4^ |
| HR-NB4 |  |  | |  |
| IRAK4 Dup | 0.332 | 5x 10^-4^ | | 5x 10^-4^ |
| CNTN5 Del | 0.394 | 10^-4^ | | 10^-4^ |
| CCPG1 | 0.69 | 10^-4^ | | 10^-4^ |
| HR-NB5 |  |  | |  |
| CNTN5-Del  SRP14 | 0.704  0.41 | 10^-5^  10^-4^ | | 10^-5^  10^-4^ |
| HR-NB6 |  |  | |  |
| NDST4 | 0.431 | 10^-4^ | | 10^-4^ |
| EWS-1 |  |  | |  |
| CDKN2A Del | 0.897 | 10^-5^ | | 10^-5^ |
| EWS-2 |  |  | |  |
| CDC117 Del | 0.412 | 10^-4^ | | 10^-5^ |
| EWS-3 |  |  | |  |
| PPP2R5 Dup | 0.38 | 10^-5^ | | 10^-5^ |
| EWS-4 |  |  | |  |
| CPEB4 Inv | 0.52 | 10^-4^ | | 10^-4^ |
| EWS-5 |  | |  |  |
| SnoU13 Del | 0.386 | | 10^-4^ | 10^-4^ |
| EWS-6 |  | |  |  |
| RASGRF2 Del | 0.33 | | 10^-4^ | 10^-4^ |
|  |  | |  |  |
|  |  | |  |  |

**Table 5.** Linear regression analysis of HR-NB and EWS specific mRNA in bone marrow spike-in experiments. (a) Mean expression values of *TH, PHOX2B* and *DCX* mRNA transcripts from 6 patients were plotted against the respective spike-in dilutions. (b) Mean expression values of *EWSR1-FLI* and *EWSR1-ETV1* mRNA from patient tumors (n=3 and n=2, respectively) were plotted against the serial dilutions of bone marrow spike-ins.

**(a)**

| **mRNA** | **r** | **95% CI** | **R^2^** | **P value** |
| --- | --- | --- | --- | --- |
| *TH* | 0.9961 | 0.9401-0.9998 | 0.9923 | 0.0003 |
| *PHOX2B* | 0.9873 | 0.8146-0.9992 | 0.9873 | 0.0017 |
| *DCX* | 0.8335 | -0.1844-0.9887 | 0.6946 | 0.0795 |

**(b)**

| **mRNA** | **r** | **95% CI** | **R^2^** | **P value** |
| --- | --- | --- | --- | --- |
| *EWSR1-FLI1* | 0.9999 | 0.9984-1.000 | 0.9998 | <0.0001 |
| *EWSR1-ETV1* | 0.9890 | 0.8371-0.9993 | 0.9781 | 0.0014 |
