# Supplementary figures and images for: Whole-genome sequencing facilitates patient-specific quantitative PCR-based minimal residual disease monitoring in acute lymphoblastic leukaemia, neuroblastoma and Ewing sarcoma
Source: Br J Cancer. 2021 Sep 1;126(3):482–91. doi: 10.1038/s41416-021-01538-z (PMC8810788; doi:10.1038/s41416-021-01538-z)

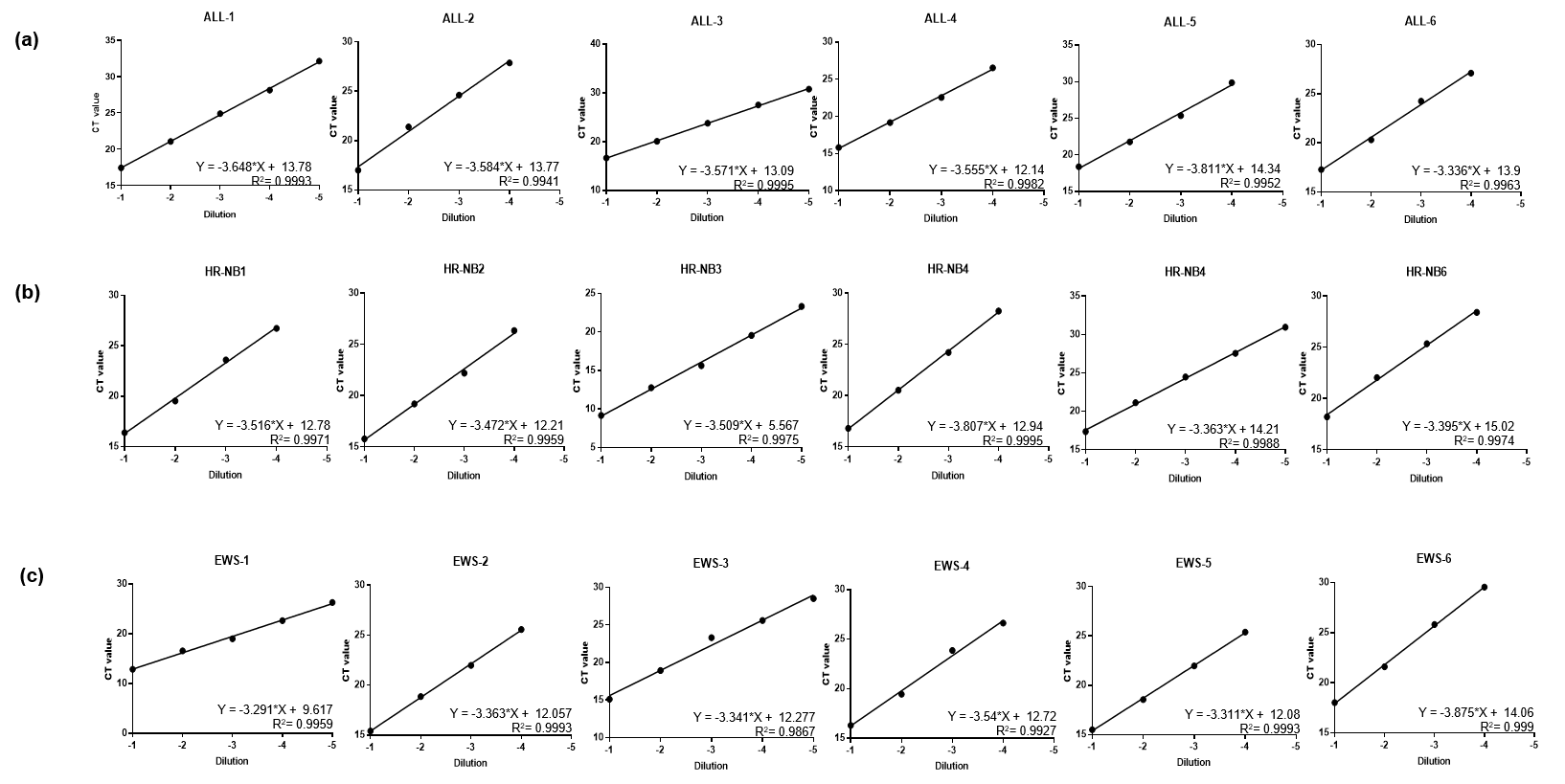

Supplement: Supplementary file 2 — Supplemental Figure 1 [file 41416_2021_1538_MOESM2_ESM.tif]

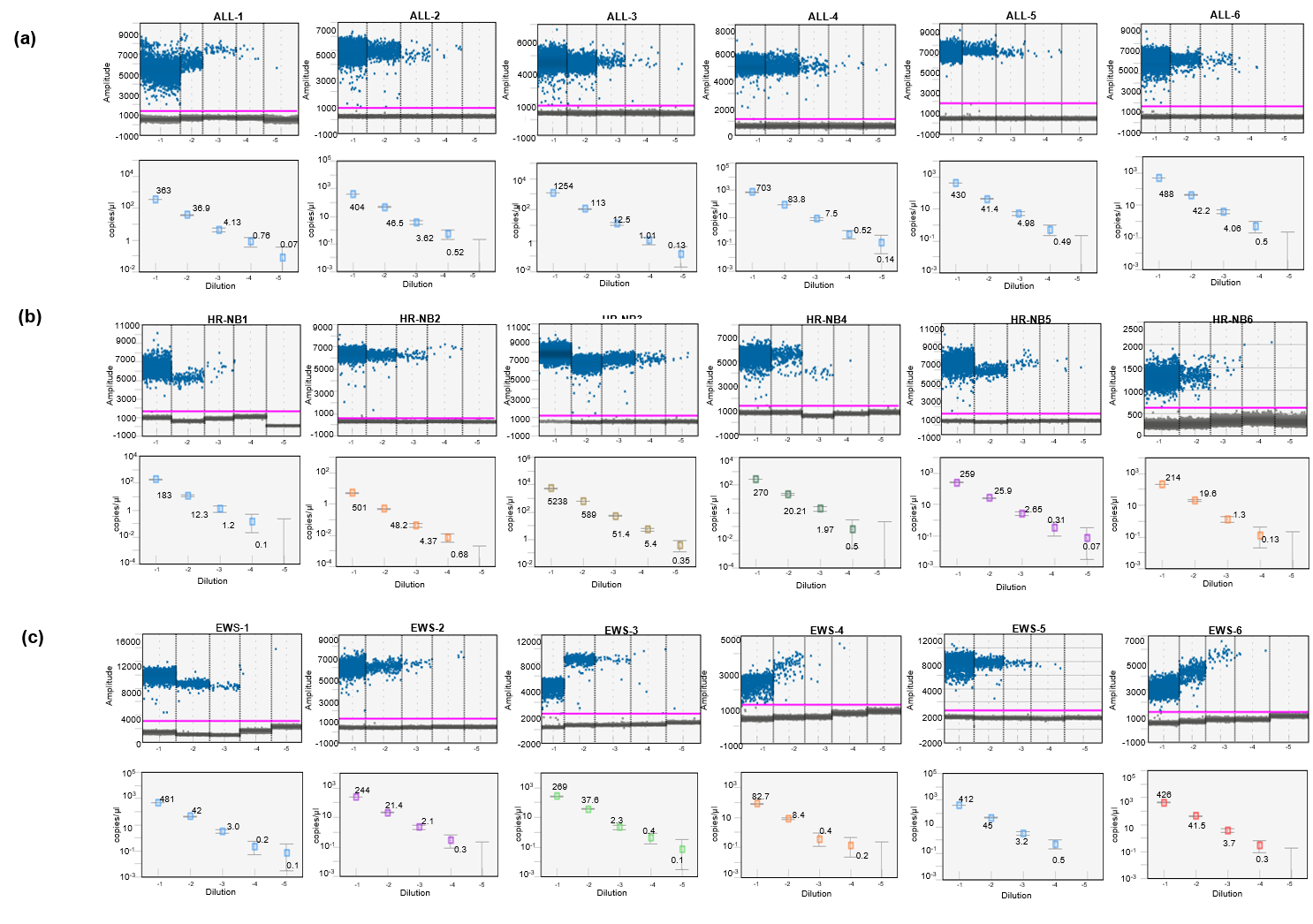

Supplement: Supplementary file 3 — Supplemental Figure 2 [file 41416_2021_1538_MOESM3_ESM.tif]

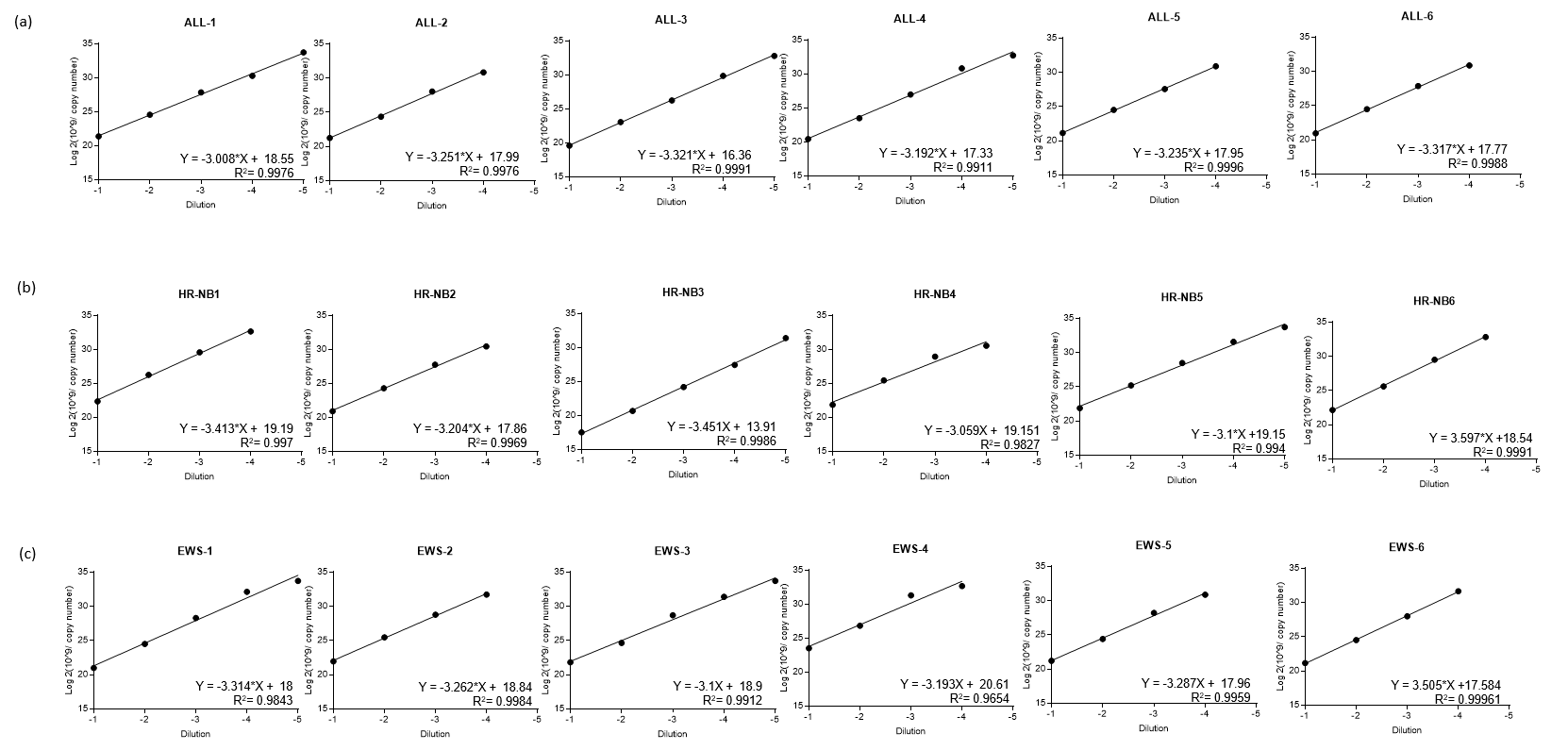

Supplement: Supplementary file 4 — Supplemental Figure 3 [file 41416_2021_1538_MOESM4_ESM.tif]
